# A Neutral Thermostable *β*-1,4-Glucanase from *Humicola insolens* Y1 with Potential for Applications in Various Industries

**DOI:** 10.1371/journal.pone.0124925

**Published:** 2015-04-24

**Authors:** Xinxin Xu, Jinyang Li, Wei Zhang, Huoqing Huang, Pengjun Shi, Huiying Luo, Bo Liu, Yuhong Zhang, Zhifang Zhang, Yunliu Fan, Bin Yao

**Affiliations:** 1 Biotechnology Research Institute, Chinese Academy of Agricultural Sciences, Beijing 100081, China; 2 Key Laboratory of Feed Biotechnology of the Ministry of Agriculture, Feed Research Institute, Chinese Academy of Agricultural Sciences, Beijing 100081, China; Weizmann Institute of Science, ISRAEL

## Abstract

We cloned a new glycoside hydrolase family 6 gene, *Hicel6C*, from the thermophilic fungus *Humicola insolens* Y1 and expressed it in *Pichia pastoris*. Using barley *β*-glucan as a substrate, recombinant HiCel6C protein exhibited neutral pH (6.5) and high temperature (70°C) optima. Distinct from most reported acidic fungal endo-*β*-1,4-glucanases, HiCel6C was alkali-tolerant, retaining greater than 98.0, 61.2, and 27.6% of peak activity at pH 8.0, 9.0, and 10.0, respectively, and exhibited good stability over a wide pH range (pH 5.0−11.0) and at temperatures up to 60°C. The *K*_m_ and *V*_max_ values of HiCel6C for barley *β*-glucan were 1.29 mg/mL and 752 μmol/min·mg, respectively. HiCel6C was strictly specific for the *β*-1,4-glucoside linkage exhibiting activity toward barley *β*-glucan, lichenan, and carboxy methylcellulose sodium salt (CMC-Na), but not toward laminarin (1,3-*β*-glucan). HiCel6C cleaved the internal glycosidic linkages of cellooligosaccharides randomly and thus represents an endo-cleaving enzyme. The predominant product of polysaccharide hydrolysis by HiCel6C was cellobiose, suggesting that it functions by an endo-processive mechanism. The favorable properties of HiCel6C make it a good candidate for basic research and for applications in the textile and brewing industries.

## Introduction

Cellulose, which consists of glucose monomer units linked by *β*-1,4-glucosidic bonds, is the most abundant carbohydrate on Earth and is considered to be a major renewable energy source [1]. Celluloses can be converted to valuable products such as biofuels, chemicals, and animal feed [2]. Due to its highly complex structure, the complete hydrolysis of cellulose requires the synergistic action of three categories of cellulases. Endo-1,4-*β*-d-glucanases (EC3.2.1.4) attack amorphous regions on soluble and insoluble 1,4-*β*-glucan substrates in a random manner to generate oligosaccharides and new chain ends, exo-1,4-*β*-d-glucanases (i.e. cellobiohydrolase, EC 3.2.1.74 and EC3.2.1.91) release cellobiose residues from the reducing or nonreducing ends of the glucan, while *β*-d-glucosidases (EC 3.2.1.21) degrade cellobiose to release glucose [3, 4].

Endo-1,4-*β*-glucanases are crucial enzymes in cellulose degradation for biomass conversion and for various industrial processes used in the textile, brewing and wine, animal feed, laundry, and pulp and paper industries [5]. Based on similarities in the amino acid sequences and structures of the proteins, endo-*β*-1,4-glucanases are classified into 11 glycoside hydrolase (GH) families designated GH5, 6, 7, 8, 9, 12, 44, 45, 48, 51, and 74 [6]. The GH6 family includes both endoglucanases (E.C3.2.1.4) and cellobiohydrolases (E.C3.2.1.91) both of which hydrolyze cellulose in a predominantly endo or processive manner from the non-reducing end to generate cellooligosaccharide or cellobiose (http://www.cazy.org).

Neutral endo-glucanases are favored by the textile industry for biostoning of indigo-dyed denim fabric because they have less aggressive effects on the fabric and have superior low backstaining properties [7]. Because of their high reaction rate and excellent stability at high temperatures, thermophilic endo-glucanases present an outstanding option for the brewing industry to improve the brewing process at elevated temperatures. However, most commercially available cellulases are derived from mesophilic filamentous fungi such as *Trichoderma* spp., *Penicillium* spp., and *Aspergillus* spp. and have acidophilic properties [8]. Therefore, novel endo-glucanases with high efficiency, neutral activity, and good thermostability are in great demand.

Filamentous fungi of the *Humicola* spp. are excellent producers of neutral thermostable cellulases for industrial applications [9–12]. Thermophilic *Humicola insolens* Y1 can produce a variety of GH enzymes with neutral pH and high temperature optima [13–15]. In this study, we cloned a novel GH6 gene (*Hicel6C*) from *H*. *insolens* Y1, expressed it in *Pichia pastoris*, and determined the biochemical properties of the recombinant HiCel6C protein. Compared to other reported fungal endo-glucanases, HiCel6C is thermophilic and alkali-tolerant and has potential for applications in the textile and brewing industries.

## Materials and Methods

### Strains, plasmids, media, and chemicals

*Humicola insolens* Y1 CGMCC 4573 was cultured routinely in wheat bran medium at 42°C as described previously [13]. *Escherichia coli* Trans1-T1 (TransGen, China) was cultivated in Luria-Bertani (LB) medium at 37°C for gene cloning and sequencing. *Pichia pastoris* GS115 (Invitrogen, USA) was cultivated in yeast peptone dextrose (YPD) medium at 30°C and used for gene expression. The plasmids pEASY-Blunt (TransGen) and pPIC9 (Invitrogen) were used as cloning and expression vectors, respectively. Substrates including barley *β*-glucan, lichenan, laminarin, birch wood xylan, 4-nitrophenyl *β*-d-cellobioside (*p*NPC), 4-nitrophenyl *β*-d-glucopyranoside (*p*NPG), and carboxymethyl cellulose sodium salt (CMC-Na) were purchased from Sigma (USA). A DNA purification kit, DNA polymerase, and restriction endonucleases were purchased from TaKaRa (Japan). T4 DNA ligase was purchased from Promega.

Minimal dextrose (MD) medium, buffered glycerol complex (BMGY) medium, and buffered methanol complex (BMMY) medium were prepared according to the manual of the *Pichia* Expression Kit (Invitrogen).

### Cloning of an *Hicel6C* cDNA

Total RNA was extracted from *H*. *insolens* Y1 using TRIzol Reagent (Invitrogen) after 48 h of cultivation in wheat bran medium. First-strand cDNA was synthesized using a PrimeScript RT reagent kit (TaKaRa). The glucanase encoding gene, *Hicel6C*, was identified in the genome sequence of *H*. *insolens* Y1 (whole genome sequencing in progress). An *Hicel6C* cDNA incorporating a C-terminal His_6_-tag coding sequence was generated using the specific primers Hicel6CF 5′-GGGAATTCGCTCCCAGCCCCAAGAGC-3′ (*Eco*RI site underlined) and Hicel6CR 5′- GCGGCCGCTTAGTGGTGGTGGTGGTGGTGCCAGAACTTGAAGATGG-3′ (*Not*I site underlined) and cloned into the vector pEASY-Blunt for sequencing.

### Sequence analysis

The Hicel6C protein sequence translated from the gene sequence was analyzed using FGENESH software (http://linux1.softberry.com/berry.phtml). An amino acid sequence alignment was analyzed using blastp software (http://www.ncbi.nlm.nih.gov/). Multiple sequence alignments were performed using the ClustalW software (http://www.ebi.ac.uk/clustalW/). A signal peptide sequence was predicted using SignalP 4.1 Server software (http://www.cbs.dtu.dk/services/SignalP/). Potential *N*-glycosylation sites were predicted using the online NetNglyc server (http://www.cbs.dtu.dk/services/NetNGlyc/). Protein functional analysis was performed using the InterProScan software (http://www.ebi.ac.uk/Tools/InterProScan/). Homology modeling and calculations of protein ionization and electrostatic potentials were performed using the Accelrys Discovery Studio 2.5 software with Cel6A (1BVW_A) and Cel6B (1DYS_A) from *H*. *insolens* serving as templates.

### Heterologous expression in *P*. *pastoris*

The *Hicel6C* cDNA sequence lacking the signal peptide coding sequence was digested with *Eco*RI and *Not*I and ligated into the corresponding sites of the pPIC9 vector to create an in-frame fusion with the *α*-factor signal peptide yielding pPIC9-*Hicel6C*. The recombinant plasmid was linearized using *Bgl*II and transformed into *P*. *pastoris* GS115 competent cells by electroporation using a Gene PulserX cell Electroporation System (Bio-Rad, USA). Positive transformants were cultured on MD plates and grown for 1–2 days at 30°C until single colonies appeared. These colonies were then transferred to 5 mL of BMGY medium and grown at 30°C for 2 d. The cells were collected by centrifugation and resuspended in 1 mL of BMMY medium containing 0.5% methanol for induction. Following induction, the culture supernatant was collected by centrifugation (12,000 × *g*, 4°C, 10 min) for use in a glucanase activity assay with barley *β*-glucan as the substrate. The positive transformant exhibiting the highest endo-glucanase activity was selected for fed-batch-mode fermentation in a 3-L fermenter containing 2 L of growth medium. The entire procedure was carried out according to the Invitrogen *Pichia* Expression Kit manual with some modifications. The positive transformant was first grown in a 250-mL flask containing 50 mL of yeast peptone dextrose medium at 30°C with agitation at 220 rpm for 48 h followed by overnight growth in a 1-L flask containing 200 mL of the same medium at 30°C and 220 rpm. Then the entire culture was transferred into a 3-L fermenter containing 2 L of basal salt medium with PMT1 trace salts solution and grown at 30°C and pH 6 with agitation at 1000 rpm and an aeration rate of 1.5 vvm. A glucose-fed batch phase was activated by addition of 25% glucose in PMT1 solution at 36 mL/h/L for 4 h when the glucose in the medium was completely consumed. A mixture of glucose and methanol (8:1) was then added at a rate of 9 mL/h/L to acclimatize the cells to methanol. The final methanol-fed batch phase was initiated for induction and expression of the recombinant protein for 108 h. The glucose was consumed completely and the dissolved oxygen level was maintained above 20% during the process. During induction/expression, the glucanase activity in the culture supernatant was assayed at multiple time intervals.

### Purification and identification of recombinant HiCel6C

The culture supernatant was collected by centrifugation (6000 × *g*, 4°C, 20 min) to remove cell debris and undissolved materials followed by concentration through a Vivaflow 200 ultrafiltration membrane with a 10-kDa molecular weight cut-off (Vivascience, Germany). The crude enzyme was loaded onto a His-Trap Sepharose XL FPLC column (Amersham Pharmacia, Sweden) pre-equilibrated with NTA0 buffer (20 mM Tris-HCl, pH 7.6, 0.5 M NaCl, 10% glycerol) and eluted using a linear gradient of imidazole (0.0–0.5 M) in NTA0 buffer at a flow rate of 3.0 mL/min. Fractions with high enzymatic activity were collected. The purity of the protein was determined by SDS-PAGE and staining with Coomassie Brilliant Blue G-250. For identification, the protein band was excised from the gel, digested with trypsin, and sequenced using liquid chromatography/electrospray ionization tandem mass spectrometry (LC-ESI-MS/MS) at the Institute of Apicultural Research, Chinese Academy of Agricultural Sciences. To determine the protein concentration, a Bradford assay was used with bovine serine albumin as the standard. To remove *N*-glycosylation, purified recombinant HiCel6C (2 μg) was treated with 500 U of endo-*β*-*N*-acetylglucosaminidase H (Endo H) for 2 h at 37°C according to the supplier's instructions (New England Biolabs, USA) and analyzed by SDS-PAGE. The native molecular weight (MW) of HiCel6C was determined by size exclusion chromatography using a 3-mL elution volume and a Superdex 200 exclusion column (Amersham Pharmacia, Sweden). The column was equilibrated and eluted with appropriate buffer (20 mM Tris-HCl, 300 mM NaCl, pH 7.2) at a flow rate of 0.2 mL/min and calibrated with lysozyme from chicken egg white (MW = 14,300), *α*-chymotrypsinogen A from bovine pancreas (MW = 24,500), albumin egg (MW = 45,000), and albumin bovine V (MW = 67,000). The apparent MW of HiCel6C was calculated from the calibration curve of log (MW) vs. elution volume.

### Glucanase activity assay

Glucanase activity was determined using the 3,5-dinitrosalicylic acid (DNS) method [16]. The standard assay system consisted of 100 μL of appropriately diluted enzyme and 900 μL of 100 mM Na_2_HPO_4_-citric acid (pH 6.5) containing 1.0% (w/v) barley *β*-glucan at 70°C for 10 min. The reaction was terminated by the addition of 1.5 mL of DNS reagent and then boiled for 5 min. When the reaction mixture cooled to room temperature, the absorbance at 540 nm was determined. One unit of enzyme activity was defined as the amount of enzyme required to release 1 μmol of reducing sugar per minute under the above conditions. Glucose was used as the standard.

### Biochemical characterization of purified recombinant glucanase

Barley *β*-glucan was used as the substrate for biochemical characterization of purified recombinant HiCel6C. The optimal pH of the purified recombinant HiCel6C was determined in various buffers with pH values ranging from 3.0 to 11.0 at 70°C for 10 min. The pH stability of HiCel6C was determined by measuring the residual enzymatic activity under standard conditions (pH 6.5, 70°C, and 10 min) after incubation at 37°C for 1 h at various pH values of 2.0–12.0. The buffers used were 100 mM Na_2_HPO_4_-citric acid (pH 2.0–8.0), 100 mM Tris-HCl (pH 8.0–9.0), and 100 mM glycine-NaOH (pH 9.0–12.0).

The optimal temperature for HiCel6C activity in the temperature range 40–90°C was determined by measuring activity in 100 mM Na_2_HPO_4_-citric acid (pH 6.5) for 10 min. The thermostability of the enzyme was determined by measuring the residual activity under standard conditions after incubation at 60, 65, and 70°C for various time periods.

To study the effects of metal ions and chemical reagents on the activity of purified recombinant HiCel6C, various metal ions (KCl, NaCl, CaCl_2_, CoCl_2_, NiCl_2_, CuSO_4_, MgSO_4_, MnSO_4_, ZnSO_4_, FeCl_3_, Pb(CH_3_COO)_2_, or AgNO_3_) or reagents (SDS, *β*-mercaptoethanol, CTAB, or EDTA) were added to the reaction system to a final concentration of 1 or 10 mM. The residual enzyme activity was determined under the standard assay conditions. An experiment without any added ion or chemical reagent was carried out as a control.

### Substrate specificity and kinetic analysis

The substrate specificity of HiCel6C was assayed at 70°C for 10 min in 100 mM Na_2_HPO_4_-citric acid (pH 6.5) containing 1.0% (w/v) barley *β*-glucan, laminarin, lichenan, birch wood xylan, Avicel, or CMC-Na or 4 mM *p*NPC and *p*NPG. The kinetic parameters *K*_m_ and *V*_max_ of HiCel6C were examined in 100 mM Na_2_HPO_4_-citric acid (pH 6.5) containing 0.2–10 mg/ml barley *β*-glucan at 70°C for 5 min. The resulting data were plotted using the Lineweaver-Burk method.

### Analysis of hydrolysis products

The reaction system containing 3 U of purified HiCel6C, 0.6 mg of cellooligosaccharide (cellobiose, cellotriose, cellotetraose, cellopentaose, or cellohexaose), and 10 mg of CMC-Na or 2 mg of barley *β*-glucan in 1 mL of 200 mM Na_2_HPO_4_-citric acid (pH 6.5) was incubated at 50°C for 20 h. For analysis of initial products, the reaction system containing 0.3 U of purified HiCel6C and 0.2 mg of cellohexaose in 1 mL of 200 mM Na_2_HPO_4_-citric acid (pH 6.5) was incubated at 70°C for 10 min. The excess enzyme was removed from the reaction system using a Nanosep centrifugal 3K device (Pall, USA). The hydrolysis products were analyzed using high-performance anion exchange chromatography (HPAEC) with a model 2500 system from Dionex (USA). Glucose and cellooligosacchardies were used as standards.

### Nucleotide sequence accession number

The nucleotide sequence of the endo-1,4-*β*-glucanase gene (*Hicel6C*) from *H*. *insolens* Y1 was deposited in the GenBank database under accession no. KM588315.

## Results and Discussion

### Cloning and sequence analysis of an *Hicel6C* cDNA from *H*. *insolens* Y1

**Fig 1:**
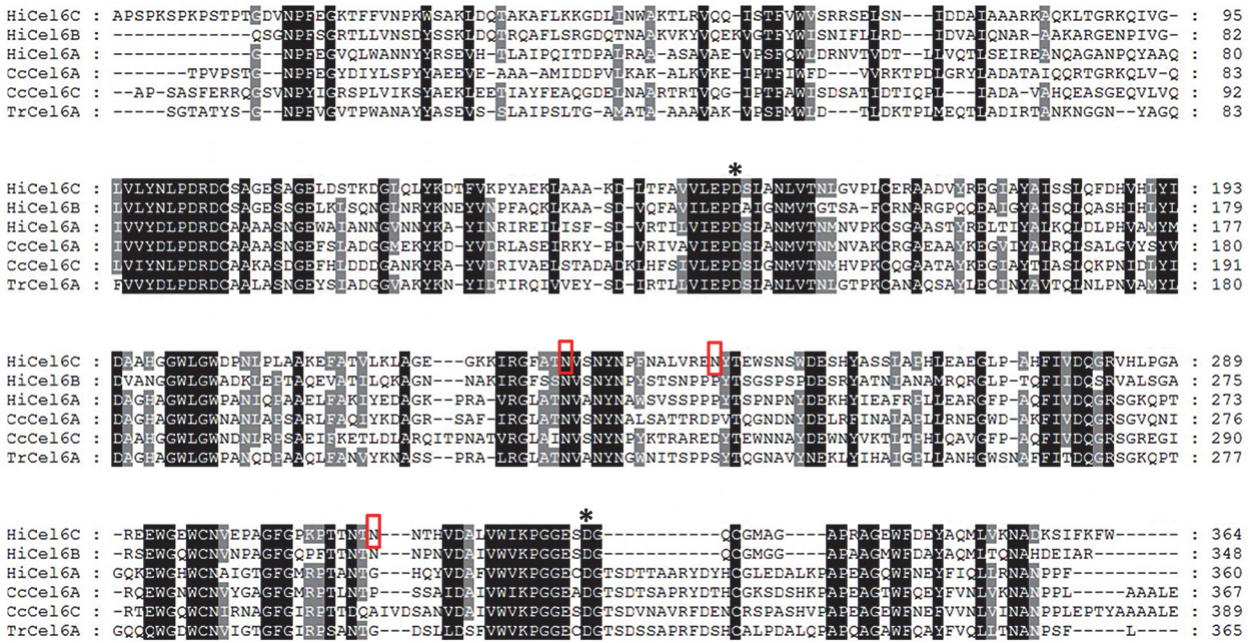
Multiple protein sequence alignment of the deduced HiCel6C sequence with five GH6 counterparts and resolved crystal structures. Sequences include the HiCel6A and HiCel6B glucanases from *H*. *insolens* (1BVW_A and 1DYS_A), CcCel6A and CcCel6C from *Coprinopsis cinerea* (3VOG_A and 3A64_A), and TrCel6A from *Trichoderma reesei* (3CBH_A). Identical and conserved residues are shaded in black and gray, respectively. Putative catalytic residues, D152 and D330, are indicated by asterisks. Potential N-glycosylation sites are boxed.

We isolated a 1149-bp, full-length *Hicel6C* cDNA from *H*. *insolens* Y1 that encoded a polypeptide 382 amino acids in length. The deduced HiCel6C protein contained a putative signal peptide at the N-terminus (residues 1–18) and a GH6 catalytic domain. The calculated molecular mass and *p*I value were estimated to be 41.7 kDa and 7.26, respectively. Further sequence analysis using the NetNGlyc Server identified three potential *N*-glycosylation sites (Asn-Xaa-Thr/Ser, where Xaa is not Pro). The deduced HiCel6C amino acid sequence shared the highest identity of 83% with a putative GH6 protein from *Chaetomium atrobrunneum* (AGV05123.1) and sequence identities of 57 and 38% with endo-*β*-1,4-glucanase (Cel6B, Q7SIG5.1) and cellobiohydrolase II (Cel6A, Q9C1S9.1), respectively, from *H*. *insolens* (Fig 1). Based on sequence alignment and homology modeling, HiCel6C has greater sequence and structural similarity to *H*. *insolens* Cel6B [17]. Both enzymes have an open N-terminal loop which is conserved among GH6 family members, but lack the C-terminal active-site-enclosing loop which is present in their cellobiohydrolase counterparts (S1 Fig). The two putative conserved catalytic residues of HiCel6C were predicted to be Asp152 and Asp330 corresponding to Asp139 and Asp316 of Cel6B.

### Expression, purification, and identification of HiCel6C

**Fig 2:**
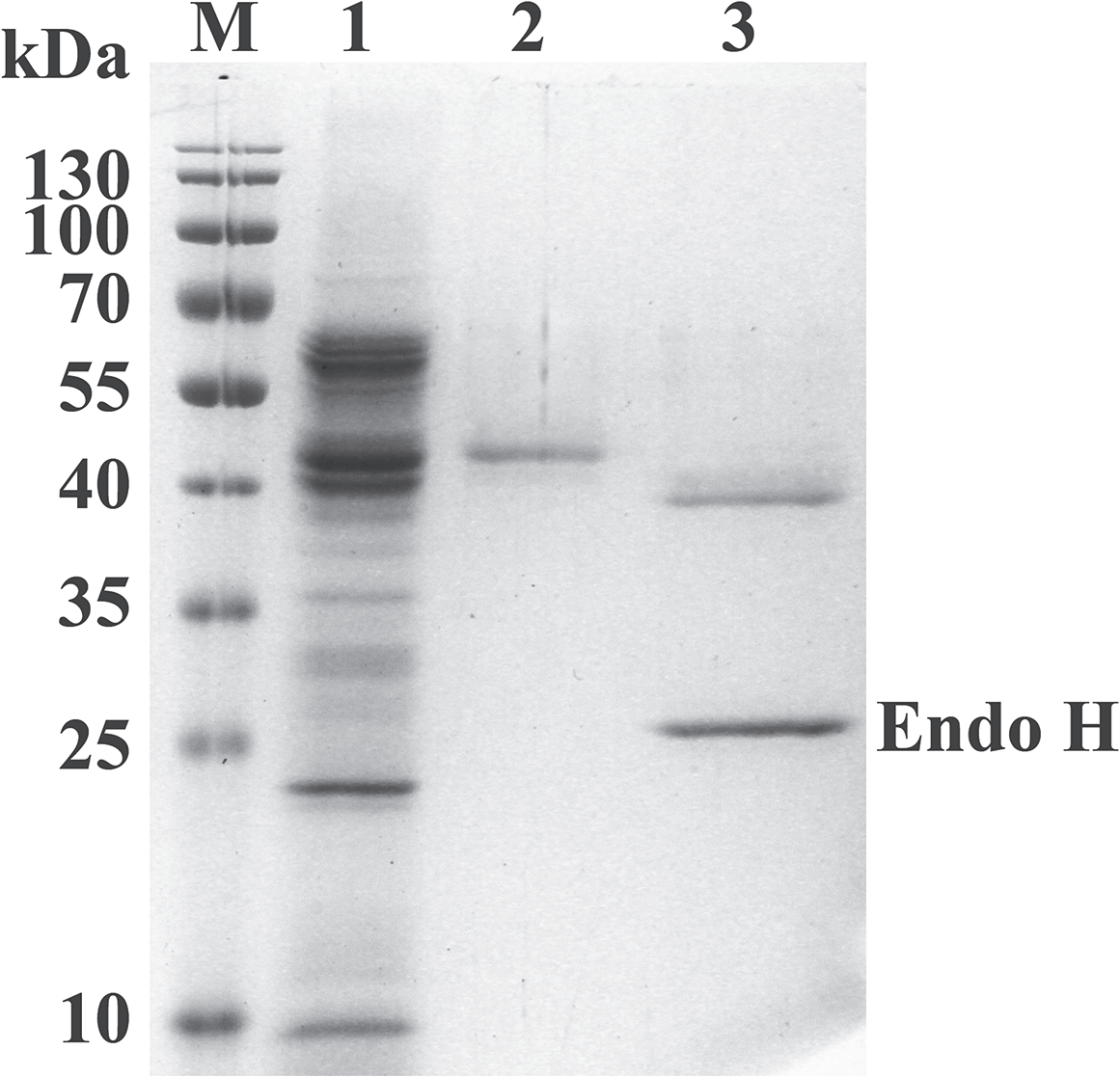
SDS-PAGE analysis of purified recombinant HiCel6C protein. Lanes: M, molecular mass markers; 1, culture supernatant of recombinant *P*. *pastoris* GS115 harboring *Hicel6C*; 2, purified recombinant HiCel6C protein; 3, HiCel6C deglycosylated by treatment with Endo H.

Using cDNA generated from strain Y1 as template and Hicel6CF and Hicel6CR primers, we amplified a fragment of the *Hicel6C* gene lacking the signal peptide coding sequence. The resulting sequence was digested with *Eco*RI and *Not*I and ligated into the vector pPIC9. The recombinant plasmid pPIC9-*Hicel6C* was integrated into the chromosome of *P*. *pastoris* GS115. The transformant exhibiting the highest endo-glucanase activity was selected for high-cell-density fermentation in a 3-L fermenter. Recombinant HiCel6C protein was purified to electrophoretic homogeneity by Ni-NTA affinity chromatography (S2 Fig). The yield of HiCel6C was approximately 200.2 mg/L. The purified fractions from the fifth and sixth collection tubes, which had the highest protein concentrations (S2 Fig), were combined for further activity analysis. Size exclusion chromatography revealed that HiCel6C eluted as a single peak with a MW of approximately 36.3 kDa, suggesting that HiCel6C was monomeric in solution (S3 Fig). The lower apparent MW after size exclusion chromatography than the calculated molecular weight of 41.7 kDa may be due to nonglobular folding of the protein. Purified HiCel6C migrated as a single band of ~50.0 kDa on SDS-PAGE which was greater than its calculated molecular weight. After deglycosylation with Endo H, the molecular mass decreased by ~8 kDa (Fig 2). *N*-glycosylation often occurs when proteins are expressed heterologously in *P*. *pastoris* [18]. The observed variation in apparent molecular mass of HiCel6C can be ascribed to glycosylation of its three putative *N*-glycosylation sites.

To identify the purified protein, seven peptides, KSPKPSTPTGDVNPFEGK, KQIVGLVLYNLPDR, DGLQLYKDTFVKPYAEK, PNLPLAAKEFATVLK, GFATNVSNYNPFNALVR, VHLPGAR, and AGEWFDEYAQMLVKNADKSIFK, obtained by LC-ESI-MS/MS were compared to the deduced HiCel6C amino acid sequence. The complete match of these sequences indicated that the purified protein was in fact recombinant HiCel6C.

### Characterization of purified recombinant HiCel6C

**Fig 3:**
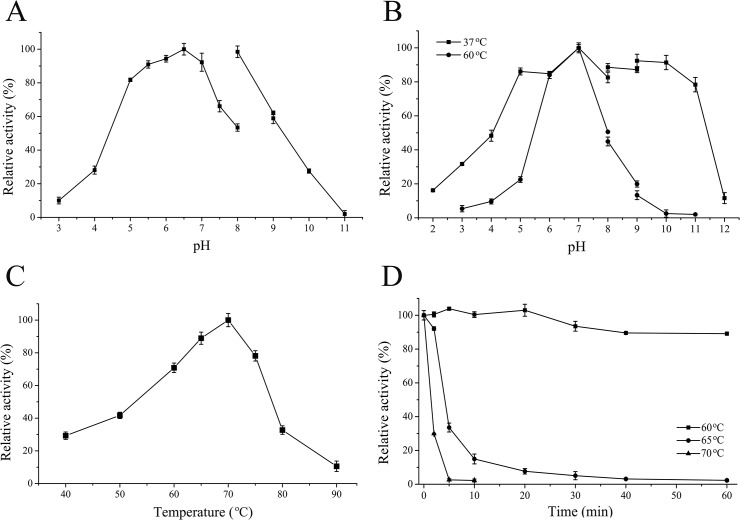
Characterization of purified recombinant HiCel6C. (A) Effect of pH on *β*-1,4-glucanase activity. (B) Effect of pH on the stability of *β*-1,4-glucanase activity. (C) Effect of temperature on *β*-1,4-glucanase activity. (D) Thermostability of purified recombinant HiCel6C. Buffers used were 100 mM Na_2_HPO_4_-citric acid (pH 2.0–8.0), 100 mM Tris-HCl (pH 8.0–9.0), and 100 mM glycine-NaOH (pH 9.0–12.0).

Most fungal endo-β-1,4-glucanases—such as endoglucanase II from *Trichoderma reesei* [19], EglA from *Aspergillus niger* VTCC-F021 [20], and EgG5 from *Phialophora* sp. G5 [21]—have an acidic pH optimum (pH 4.0–6.0). However, the cellulases and hemicellulases from *Humicola* sp. such as rCBH1.2 from *H*. *grisea* var. *thermoidea* (pH optimum of 8.0) [22] and XynA and Man5A from *H*. *insolens* Y1 [13, 14] are alkali-tolerant. HiCel6C exhibited a similar preference for alkaline conditions with a pH optimum of 6.5 (Fig 3A) and it retained greater than 98% of its peak activity at pH 8.0 and 61.2 and 27.6% of peak activity at pH 9.0 and 10.0, respectively. The enzyme was also stable over a wide pH range of 5.0–11.0 at 37°C with retention of greater than 80% of activity after 1 h of incubation. When the temperature was increased to 60°C, HiCel6C activity was stable only in the pH range 5.0–11.0 (Fig 3B). The profile splitting in Fig 3A and 3B was caused by buffer changes required for the assay. Due to the strength and nature of ions, enzyme activity may vary considerably in different buffer systems even at identical pH. Electrostatic potential analysis indicated that the number of basic residues located on the HiCel6C protein surface was greater than for its acidophilic counterparts (data not shown). This characteristic may be an important factor in the high adaptability and stability of HiCel6C under alkaline conditions. Previous studies demonstrated that neutral cellulases tend to cause much less indigo backstaining and higher abrasion than acid enzymes during biostoning of denim garments [23, 24]. The excellent alkali-tolerance of neutral HiCel6C makes it particularly well suited for applications in the textile industry.

The optimal temperature for HiCel6C activity was estimated to be 70°C (Fig 3C) which is greater than generally known fungal endo-β-1,4-glucanases which have temperature optima of 50–60°C and are stable up to 50–55°C. Under high temperature conditions, *β*-1,4-glucanases from thermophilic fungi are more active and more stable than those from mesophiles [25–27]. To date, only a few fungal endo-β-1,4-glucanases, such as those from *Talaromyces emersonii* CBS394.64 [28], *Penicillium pinophilum* [2], and *Phialophora* sp. G5 [29], have been shown to have temperature optima similar to or higher than that of HiCel6C. We also investigated the thermal stability of purified HiCel6C (Fig 3D). After incubation at 60°C for 1 h, the enzyme retained greater than 90% of its initial activity. Thermostable endoglucanases are of great interest due to their potential applications in various industrial processes such as bioconversion of biomass into fermentative products, improvement of barley malting in brewing, and modification of the coarse mechanical pulp and reduction of the amount of chlorine used for bleaching in the pulp and paper industry.

The effects of metal ions and chemical reagents on HiCel6C activity were determined at concentrations of 1 and 10 mM (Table 1). Most metal ions and chemicals had no significant effect on HiCel6C activity with the exceptions of CTAB and Cu^2+^ at 10 mM and Mn^2+^ at 1 and 10 mM. HiCel6C exhibited significant resistance to SDS at concentrations of 1 and 10 mM, retaining 76.3 and 31.6% of activity, respectively. Thus HiCel6C has properties that are advantageous for applications in laundry detergents and the textile industry.

**Table 1:** Effect of metal ions and chemical reagents on the *β*-1,4-glucanase activity of purified recombinant HiCel6C.

	Relative activity (%)^a^	
Metal ions and reagents	1 mM	10 mM
Control	100.0	100.0
NaCl	96.1 ± 1.0	99.8 ± 2.3
KCl	101.3 ± 1.8	106.6 ± 0.7
CaCl_2_	101.6 ± 3.3	79.4 ± 2.6
CoCl_2_	108.5 ± 2.2	81.5 ± 2.5
NiCl_2_	98.4 ± 3.7	95.8 ± 2.9
CuSO_4_	99.1 ± 3.1	30.0 ± 2.8
MgSO_4_	94.6 ± 1.7	81.0 ± 2.1
FeCl_3_	91.1 ± 4.0	54.2 ± 2.8
MnSO_4_	59.5 ± 1.8	0
ZnSO_4_	91.7 ± 1.9	91.6 ± 0.8
Pb(CH_3_COO)_2_	94.2 ± 0.5	57.8 ± 1.7
AgNO_3_	86.1 ± 2.2	77.6 ± 1.3
SDS	76.3 ± 0.1	31.6 ± 2.1
CTAB	30.6 ± 0.2	21.4 ± 1.0
EDTA	90.9 ± 3.4	70.1 ± 3.0
*β*-Mercaptoethanol	93.7 ± 2.3	83.5 ± 1.3

^a^ Values represent the means of triplicates relative to the untreated control samples.

### Substrate specificity and kinetic parameters

The highest enzymatic activity of the purified HiCel6C was exhibited against barley *β*-glucan (100.0%) followed by lichenan (60.5%) and CMC-Na (13.6%), but no activity was shown against laminarin (1,3–1,6-*β*-glucan), 4-nitrophenyl *β*-d-cellobioside (*p*NPC), or 4-nitrophenyl *β*-d-glucopyranoside (*p*NPG) (Table 2). The HiCel6C activity was strictly specific for the *β*-1,4-glucoside linkage. The higher activity of HiCel6C against barley *β*-glucan than CMC-Na may be ascribed to the fact that CMC is highly substituted with methoxy side chains which interfere with the enzyme activity [30]. Moreover, HiCel6C was inactive against Avicel, filter paper, and birch wood xylan. The inactivity against crystalline substrates such as Avicel may be ascribed to the presence of a single catalytic domain in HiCel6C without a carbohydrate-binding module.

**Table 2:** Substrate specificity of purified recombinant HiCel6C.

Substrate	Major linkage type	Specific activity (U/mg)	Relative activity (%)
Barley *β*-glucan	1,3–1,4-*β*-(Glucose)	372 ± 15	100
Lichenan	1,3–1,4-*β*-(Glucose)	225 ± 8	60.5
CMC-Na	1,4-*β*-(Glucose)	51 ± 4	13.6
Laminarin	1,3–1,6-*β*-(Glucose)	–^a^	–
Birch wood xylan	1,4-*β*-(Xylose)	–	–
Avicel	1,4-*β*-(Glucose)	–	–
*p*NPG	1,4-*β*-(Nitrophenyl)	–	–
*p*NPC	1,4-*β*-(Nitrophenyl)	–	–

^a^, not detected.

When using barley *β*-glucan as the substrate, the specific activity and *K*_m_ and *V*_max_ values of purified recombinant HiCel6C were 372 U/mg, 1.29 mg/mL, and 752 μmol/min·mg, respectively.

### Analysis of hydrolysis products

To explore the mode of action of HiCel6C, we analyzed the products of cellooligosaccharide hydrolysis by HiCel6C using high-performance anion exchange chromatography (HPAEC). HiCel6C did not degrade cellobiose, but had low activity against cellotriose to produce glucose and cellobiose (S4 Fig). In the presence of HiCel6C, cellotetraose was degraded completely with cellobiose as the main product. Under the same conditions, cellopentaose was hydrolyzed to cellobiose, cellotriose, and small amounts of glucose. The products of cellohexaose hydrolysis during the initial 10 min included cellobiose, cellotriose, cellotetraose, and a trace amount of glucose (S5 Fig). Upon further incubation, cellohexaose was hydrolyzed completely to cellobiose, cellotriose, and a small amount of glucose (S4 Fig). These results indicated that HiCel6C cuts cellooligosaccharides randomly and thus represents a typical endo-cleaving enzyme. HiCel6C provides another example of GH6 endoglucanases that lack the C-terminal active-site-enclosing loop. The C-terminal active-site-enclosing loop may be the crucial element that differentiates GH6 endo- and exo-glucanases. This hypothesis was verified by site-directed mutation of the C-terminus-proximal loop in cellobiohydrolase A of *Cellulomonas fimi*, which enhanced its endoglucanase activity [31].

**Fig 4:**
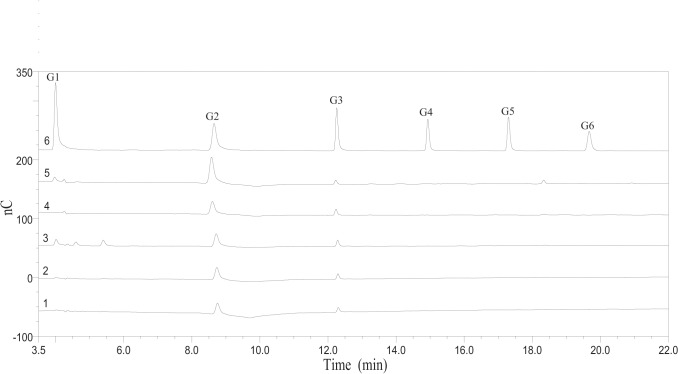
HPAEC analysis of the products of CMC-Na hydrolysis by HiCel6C. 1–5, hydrolysis products of CMC-Na after incubation with HiCel6C for 5 min, 10 min, 30 min, 1 h, and 24 h, respectively; 6, cellooligosaccharides standards. G1, glucose; G2, cellobiose; G3, cellotriose; G4, cellotetraose; G5, cellopentaose; and G6, cellohexaose.

We also examined the reaction products generated hydrolysis of barley *β* -glucan and CMC-Na by HiCel6C (Fig 4). Cellobiose was the predominant hydrolysis product of both barley β-glucan and CMC-Na followed by small amounts of glucose and cellotriose. This result provided evidence for a potential processive mechanism of HiCel6C activity similar to that of Cel5 from *Hahella chejuensis* [32], Cel5H from *S*. *degradans* [33], and EG1 from *V*. *volvacea* [34] all of which have been reported to be processive endoglucanases of the GH5 family.

## Conclusions

We identified a novel GH6 endo-*β*-1,4-glucanase gene from *H*. *insolens* Y1 and overexpressed it in *P*. *pastoris* GS115. Recombinant HiCel6C protein exhibited optimal activity at pH 6.5 and 70°C when barley β-glucan was used as substrate. HiCel6C was alkali-tolerant and remained stable over a pH range of 5.0–11.0 and at temperatures up to 60°C. Substrate specificity and hydrolysis product analysis indicated that HiCel6C is a typical *β*-1,4-glucanase with an endo-cleaving mode. These properties make HiCel6C of considerable interest for basic research and for various industrial applications, especially in the textile and brewing industries.

## Supporting Information

S1 Fig Modeled structures of the active-center loops of the *H*. *insolens* GH6 cellulases HiCel6C (blue), HiCel6B (yellow), and HiCel6A (red). (DOC)

S2 Fig Recombinant HiCel6C purified by Ni-NTA chromatography. (A) Profile of the purified protein corresponding to the collection tubes; (B) SDS-PAGE analysis of the purified protein. M: protein markers; lanes 1–5: fractions corresponding to collection tubes 4–8.
(DOC)

S3 Fig Size exclusion chromatography of purified HiCel6C. (A) Calibration curve of standard proteins showing the relationship between retention volume and the log MW of the macromolecules. (B) Profile of purified HiCel6C.
(DOC)

S4 Fig HPAEC analysis of products of cellooligosaccharide hydrolysis by HiCel6C. 1, cellohexaose; 2, cellopentaose; 3, cellotetraose; 4, cellotriose; 5, cellobiose; 6, cellooligosaccharide standards. G1, glucose; G2, cellobiose; G3, cellotriose; G4, cellotetraose; G5, cellopentaose; and G6, cellohexaose.
(DOC)

S5 Fig HPAEC analysis of the initial products of cellohexaose hydrolysis by HiCel6C. 1, cellooligosaccharides standards. G1, glucose; G2, cellobiose; G3, cellotriose; G4, cellotetraose; G5, cellopentaose; G6, cellohexaose; and 2, control reaction without enzyme; 3, products of cellohexaose hydrolysis by HiCel6C.
(DOC)
