# Supplementary material for: A Neutral Thermostable β-1,4-Glucanase from Humicola insolens Y1 with Potential for Applications in Various Industries
Source: PLoS One. 2015 Apr 24;10(4):e0124925. doi: 10.1371/journal.pone.0124925 (PMC4409357; doi:10.1371/journal.pone.0124925)
Supplement: S1 Fig — (DOC) [file pone.0124925.s001.doc]

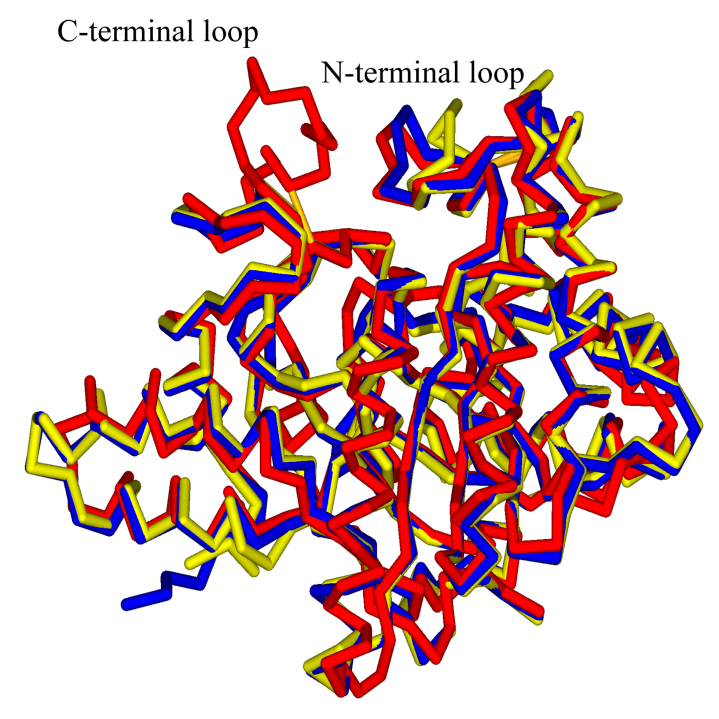


**S1 Fig. Modeled structures of the active-center loops of the *H. insolens* GH6 cellulases HiCel6C (blue), HiCel6B (yellow), and HiCel6A (red).**
