# Supplementary material for: A Neutral Thermostable β-1,4-Glucanase from Humicola insolens Y1 with Potential for Applications in Various Industries
Source: PLoS One. 2015 Apr 24;10(4):e0124925. doi: 10.1371/journal.pone.0124925 (PMC4409357; doi:10.1371/journal.pone.0124925)
Supplement: S2 Fig — (A) Profile of the purified protein corresponding to the collection tubes; (B) SDS-PAGE analysis of the purified protein. M: protein markers; lanes 1–5: fractions corresponding to collection tubes 4–8. (DOC) [file pone.0124925.s002.doc]

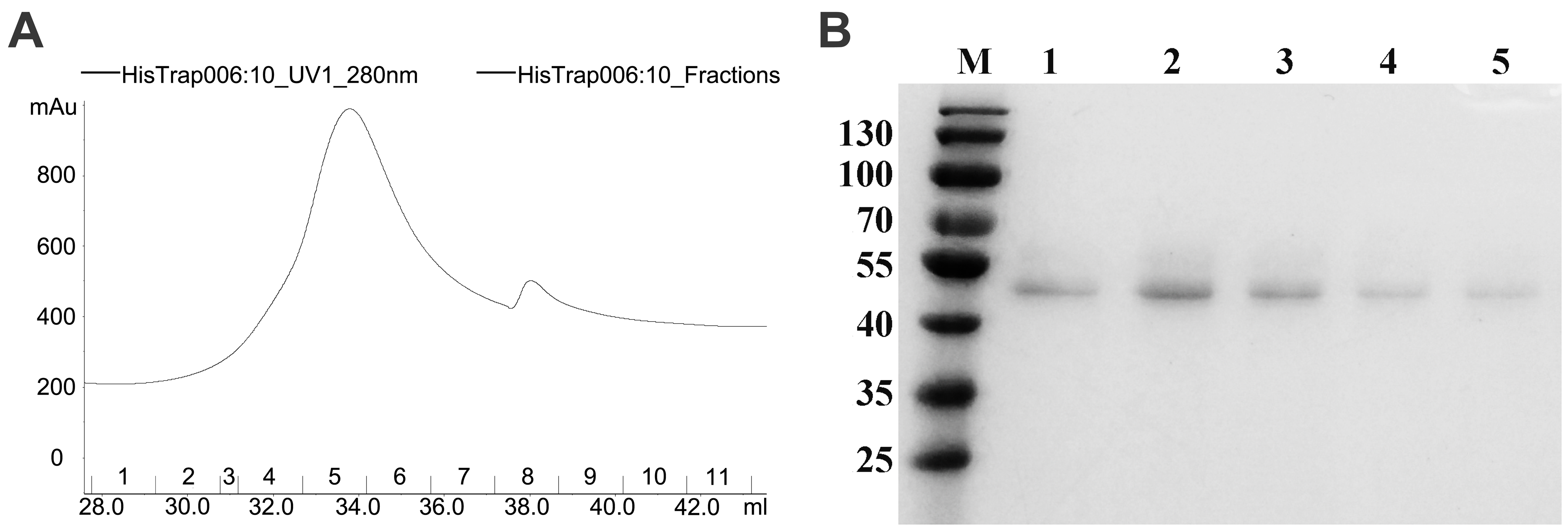


**S2 Fig. Recombinant HiCel6C purified by Ni-NTA chromatography.** (A) Profile of the purified protein corresponding to the collection tubes; (B) SDS-PAGE analysis of the purified protein. M: protein markers; lanes 1–5: fractions corresponding to collection tubes 4−8.
