# Supplementary material for: A Neutral Thermostable β-1,4-Glucanase from Humicola insolens Y1 with Potential for Applications in Various Industries
Source: PLoS One. 2015 Apr 24;10(4):e0124925. doi: 10.1371/journal.pone.0124925 (PMC4409357; doi:10.1371/journal.pone.0124925)
Supplement: S3 Fig — (A) Calibration curve of standard proteins showing the relationship between retention volume and the log MW of the macromolecules. (B) Profile of purified HiCel6C. (DOC) [file pone.0124925.s003.doc]

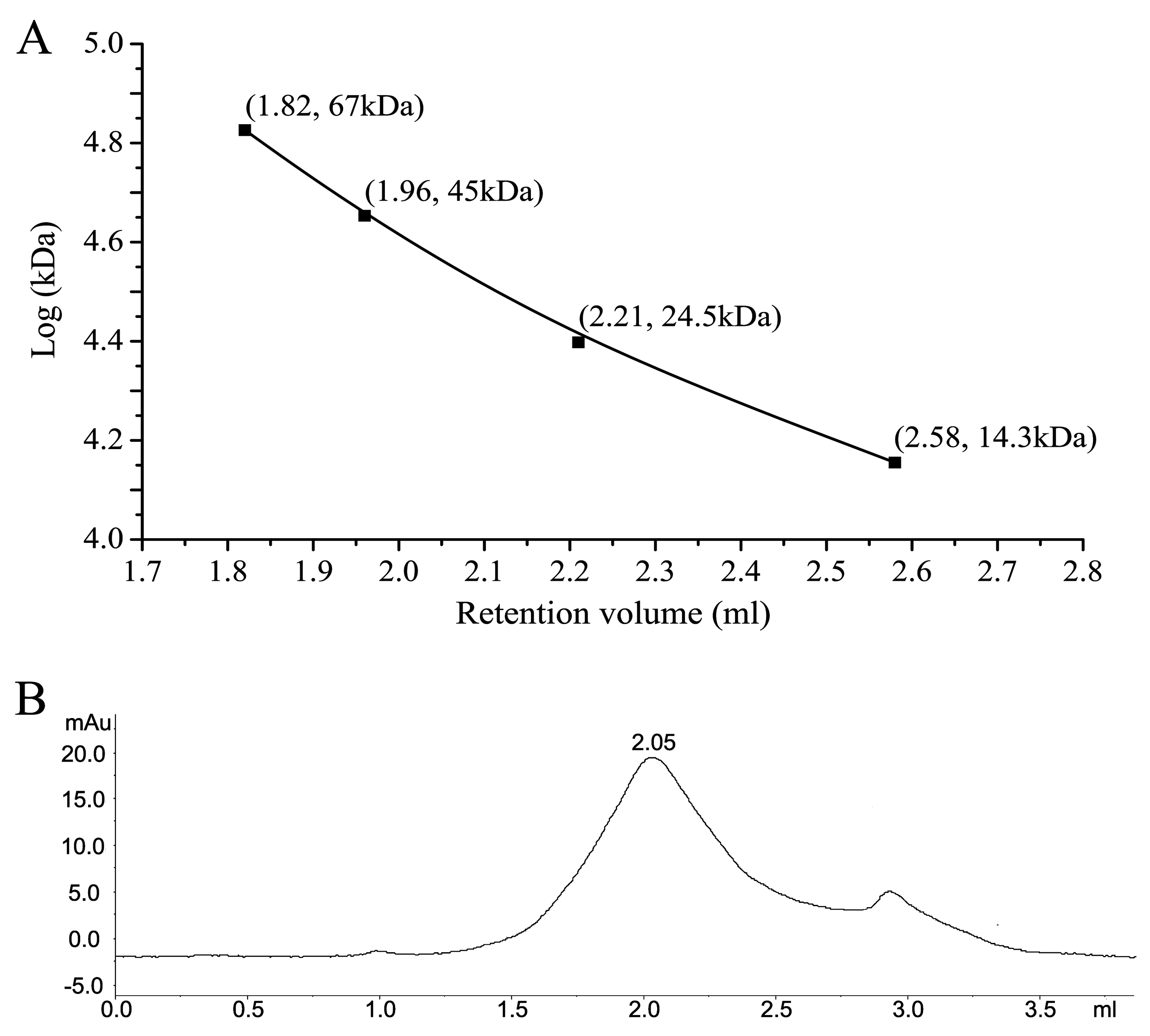


**S3 Fig. Size exclusion chromatography of purified HiCel6C.** (A) Calibration curve of standard proteins showing the relationship between retention volume and the log MW of the macromolecules. (B) Profile of purified HiCel6C.
