# Supplementary material for: A Neutral Thermostable β-1,4-Glucanase from Humicola insolens Y1 with Potential for Applications in Various Industries
Source: PLoS One. 2015 Apr 24;10(4):e0124925. doi: 10.1371/journal.pone.0124925 (PMC4409357; doi:10.1371/journal.pone.0124925)
Supplement: S4 Fig — 1, cellohexaose; 2, cellopentaose; 3, cellotetraose; 4, cellotriose; 5, cellobiose; 6, cellooligosaccharide standards. G1, glucose; G2, cellobiose; G3, cellotriose; G4, cellotetraose; G5, cellopentaose; and G6, cellohexaose. (DOC) [file pone.0124925.s004.doc]

**S4 Fig. HPAEC analysis of products of cellooligosaccharide hydrolysis by HiCel6C.** 1, cellohexaose; 2, cellopentaose; 3, cellotetraose; 4, cellotriose; 5, cellobiose; 6, cellooligosaccharide standards. G1, glucose; G2, cellobiose; G3, cellotriose; G4, cellotetraose; G5, cellopentaose; and G6, cellohexaose.
