# Supplementary material for: A Neutral Thermostable β-1,4-Glucanase from Humicola insolens Y1 with Potential for Applications in Various Industries
Source: PLoS One. 2015 Apr 24;10(4):e0124925. doi: 10.1371/journal.pone.0124925 (PMC4409357; doi:10.1371/journal.pone.0124925)
Supplement: S5 Fig — 1, cellooligosaccharides standards. G1, glucose; G2, cellobiose; G3, cellotriose; G4, cellotetraose; G5, cellopentaose; G6, cellohexaose; and 2, control reaction without enzyme; 3, products of cellohexaose hydrolysis by HiCel6C. (DOC) [file pone.0124925.s005.doc]

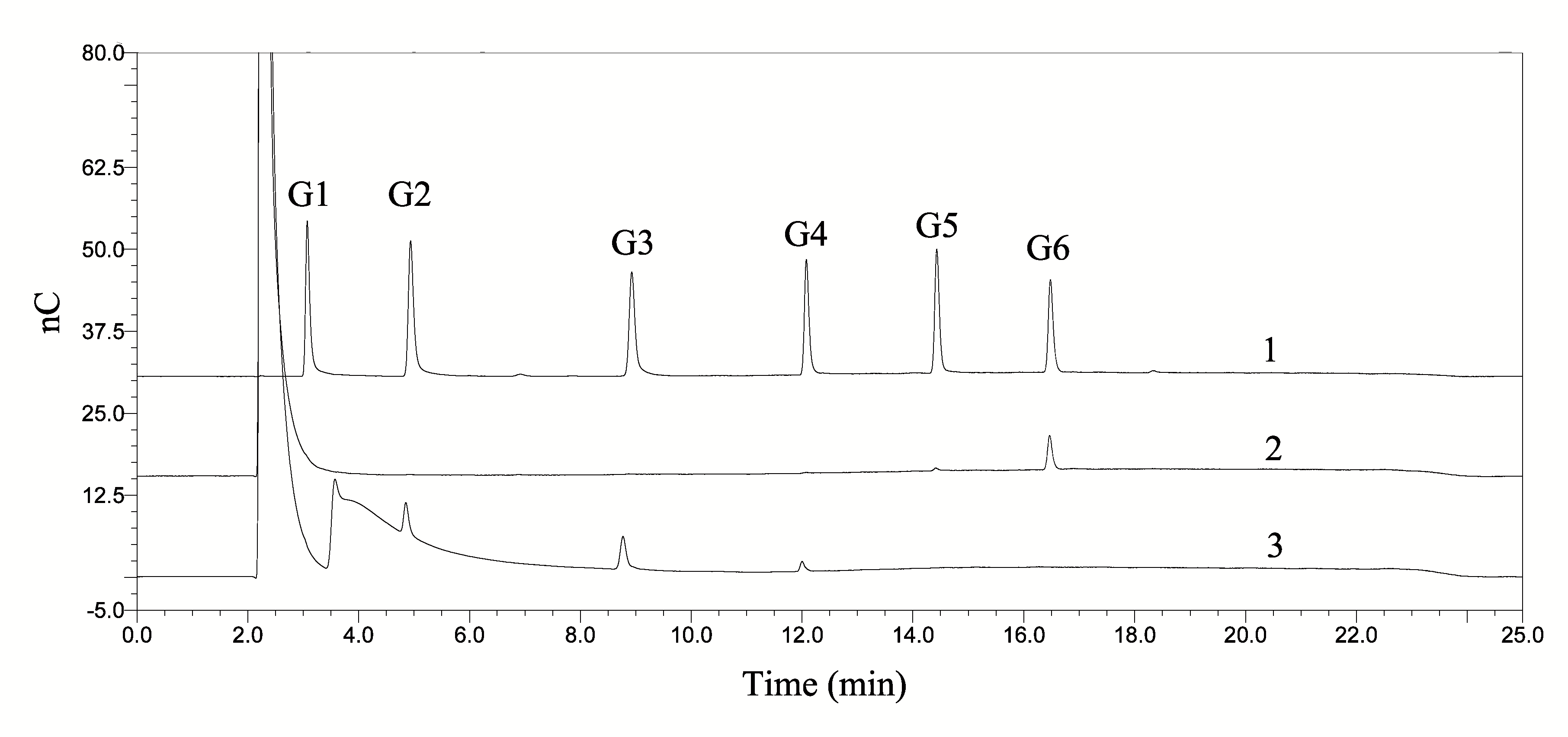


**S5 Fig. HPAEC analysis of the initial products of cellohexaose hydrolysis by HiCel6C.** 1, cellooligosaccharides standards. G1, glucose; G2, cellobiose; G3, cellotriose; G4, cellotetraose; G5, cellopentaose; G6, cellohexaose; and 2, control reaction without enzyme; 3, products of cellohexaose hydrolysis by HiCel6C.
